# The Case for the Ward Dressing Room

**Published:** 1913-04-05

**Authors:** 


### PROBLEMS IN ADMINISTRATIVE MEDICINE.

(Carefully thought-out Contributions to this Section will be welcome.)

### IV. The Case for the Ward Dressing Room.

A study of the plans of newer hospitals in this country reveals one interesting fact. Modern hospitals abroad make provision for a ward "treatment or dressing room." This is a small room opening out of the ward, or out of the annexe corridor, but so placed, at all events, that it is readily accessible from the ward. This room serves as a dressing station for cases that need special attention. In comparatively few of our hospitals is such provision made. In our large London hospitals, for instance, we may take the case of a patient who has just been operated upon for appendicular abscess. The patient is in a surgical ward; he has a large drainage tube (we will suppose that he has not been sutured immediately after evacuation of the abscess, but that drainage has been employed, as is the custom in by far the majority of cases), in which is inserted a length of gauze. This gauze has to be taken out and renewed every twenty-four hours for several days; probably also the wound has to be gently irrigated with some lotion or antiseptic solution. The ordinary method of procedure in such cases is as follows: The nurse surrounds the bed by screens. The instruments for the dressing are sterilised in the ward steriliser and are taken to the bedside in covered trays. For the moment the space within the enclosure of screens is transformed into a miniature operating theatre. All this takes place in the presence of a number of sick patients, some of whom have already been operated upon, others who are waiting for operation on the same or the following day. If the patient is a child, the preliminaries may put him in such a state of agitation that the whole ward is disturbed; even in the case of an adult, especially if he or she be nervous and the dressing is painful, the actual replacement of the drains may cause involuntary protests in the shape of cries or groans. While the actual operation is necessarily hidden from the interested audience by the screens, these flimsy walls cannot shut out the cries or groans of the patient. Ward dressing, no matter how well conducted, how gently or how carefully, is always a cause of disturbance to the ward wherein it takes place. That is the main reason for the provision of a separate dressing room.

It may be said that the procedure outlined above is rarely followed. We know that it is a fact that in many cases such dressings as described above are done in the theatre. The patient is wheeled out of the ward and taken to the operating room where the dressing is done with the usual aseptic precautions and care that are taken in the case of a major operation. But this ideal is not always attainable in a hospital ward. For in the first place the operating theatre is not always available; it has to be prepared for operations later on in the day. Secondly, in hospitals of moderate size there is usually only one theatre or at most two. If there are two one can easily be used for septic dressings, but where there is only one theatre it is questionable whether it is justifiable to take septic cases for dressing into it.

We are of opinion that hospital committees should make a point of insisting that a treatment room should be considered by the architect when designing his ward plans. At present it is lamentable that so many wards are constructed without providing for such a room. Its value is, indeed, incalculable. We have referred to the septic appendix case, but that type of case does not stand alone. Our contention is—and we feel sure that hospital workers will cordially agree with us in this—that sick patients ought not to be subjected to the psychical excitement which invariably follows when they are made to witness the preliminaries for an operation. The sight of instruments, and the mere fact that an operation, however trivial it may seem to the nursing and medical staff, is in progress in their immediate neighbourhood, however much screened it may be, are sufficient to stimulate excitement which in some cases may be dangerous and in all must be objectionable. In a children's ward the necessity for a special treatment room is even more imperative than in an adult ward. Children stand slight operations, such as surgical dressings, remarkably well on the whole, but they sometimes protest very vigorously. We recollect in our student days the case of a small boy from whom we had to remove the little collodion pad and dressing on a slight tenotomy wound. The operation, trivial in the extreme and utterly painless, was done to the accompaniment of a chorus of yells which roused sympathetic crying from three other patients in the ward. The result of that simple dressing was to be seen on several bed letters that night, when it was found necessary to prescribe opiates or sleeping draughts to patients who would otherwise, possibly, not have required such help. Every hospital worker can multiply incidences of this kind. They serve to show that the greatest care must be taken to prevent undue excitement and psychical stimulus to sick patients. We have repeatedly laid stress on the humane aspect of hospital treatment, and it is just in attention to such small points that the humanity of the hospital worker shows itself. With a special treatment room, a large part of the difficulty of dealing with surgical cases that want daily dressing is satisfactorily got rid of. Nothing is easier than to wheel the patient into the dressing room. Before his entrance everything has been made ready for the dressing; instruments and appliances are hidden as much as possible, the room is comfortably warmed and lighted, and there is all the privacy of an operating theatre without much of its accompanying impressiveness that to some patients is a source of terror and excitement. The dressing, in such circumstances, is easily and quickly done; a refractory child, for example, is much more readily dressed in the treatment room than in the ward. We have had personal experience in this matter and can vouch for the truth of the statement, although we do not pretend to explain it. Probably psychical stimulus is again the cause. In the ward the child cries because he instinctively knows that he will obtain sympathy, or there is often, perhaps, even an element of that perversity which characterised the child in "Alice" who "only does it to annoy, because he knows it teases."

In the dressing room there is no inducement to do this; he knows that the dressing has to be done and soon learns that it is done not to give him pain, but to cure or relieve him. In adult cases the subsequent pain or inconvenience that may produce involuntary cries, are the same in the ward as in the dressing room, but the patient can be kept in the dressing room for a little while after the dressing has been completed and so given an opportunity of mastering his distress before being wheeled back into the ward. In any case the provision of a dressing room conduces to the harmony and peace of the ward, and no hospital worker who has had experience of it can fail to appreciate its merits. It should equally be insisted upon in infirmaries.

